# Trimebutine Maleate Monotherapy for Functional Dyspepsia: A Multicenter, Randomized, Double-Blind Placebo Controlled Prospective Trial

**DOI:** 10.3390/medicina56070339

**Published:** 2020-07-08

**Authors:** Jannis Kountouras, Emmanuel Gavalas, Apostolis Papaefthymiou, Ioannis Tsechelidis, Stergios A. Polyzos, Serhat Bor, Mircea Diculescu, Κhaled Jadallah, Mazurek Tadeusz, Tarkan Karakan, Anna Bochenek, Jerzy Rozciecha, Piotr Dabrowski, Zeno Sparchez, Orhan Sezgin, Macit Gülten, Niazy Abu Farsakh, Michael Doulberis

**Affiliations:** 1 Second Medical Clinic, School of Medicine, Aristotle University of Thessaloniki, Ippokration Hospital, 54642 Thessaloniki, Macedonia, Greece; emmanouel_gavalas@yahoo.com (E.G.); appapaef@hotmail.com (A.P.); tsechelidis@gmail.com (I.T.); spolyzos@auth.gr (S.A.P.); doulberis@gmail.com (M.D.); 2 Department of Gastroenterology, University General Hospital of Larissa, Mezourlo, 41334 Larissa, Thessaly, Greece; 3 First Laboratory of Pharmacology, School of Medicine, Aristotle University of Thessaloniki, 54124 Thessaloniki, Macedonia, Greece; 4 Division of Gastroenterology, Ege University School of Medicine, 35330 Izmir, Turkey; serhat.bor@ege.edu.tr; 5 Gastroenterology and Hepatology Department, Clinic Fundeni Institute, 4204003 Bucharest, Romania; gastrofundeni@cmb.ro; 6 Department of Internal Medicine, King Abdullah University Hospital, 22110 Irbid, Jordan; nadamopoulos69@gmail.com (K.J.); pm2@galenika.gr (N.A.F.); 7 Medicor Centrum, ul. Jabłoskiego 2/4, 35-068 Rzeszów, Poland; medicor@medicor.pl; 8 Department of Gastroenterology, Gazi University School of Medicine, 06560 Ankara, Turkey; tkarakan@gmail.com; 9 Centrum Badawcze Wspolczesnej Terapii, 02679 Warszawa, Poland; macedonia@galenica.gr; 10 LexMedica, Rudolfa Weigla 12, Krzyki, 53114 Wrocław, Poland; j.rozciecha@lexmedica.eu; 11 Department of Rheumatology of Clinical Hospital 2, University of Rzeszow, Lwowska 60, 35-301 Rzeszow, Poland; recepcja@klinika-rzeszow.pl; 12 Third Medical Clinic, University of Medicine and Pharmacy, Croitorilor Street no.19-21, 400162 Cluj-Napoca, Romania; nbd@galenica.gr; 13 Department of Gastroenterology, Faculty of Medicine, Mersin University, 33343 Mersin, Turkey; drorhansezgin@gmail.com; 14 Department of Gastroenterology, Uludag University, 16059 Bursa, Turkey; macit@uludag.edu.tr; 15 Division of Gastroenterology and Hepatology, Medical University Department, Kantonsspital Aarau, 5001 Aarau, Switzerland

**Keywords:** trimebutine maleate, functional dyspepsia, gastrointestinal function, gastrointestinal motility

## Abstract

*Background and Objectives:* Functional dyspepsia (FD) is one of the most common functional gastrointestinal disorders; it has a great impact on patient quality of life and is difficult to treat satisfactorily. This study evaluates the efficacy and safety of trimebutine maleate (TM) in patients with FD. *Materials and Methods*: A multicenter, randomized, double-blind, placebo controlled, prospective study was conducted, including 211 patients with FD. Participants were randomized to receive TM 300 mg twice per day (BID, 108 patients) or placebo BID (103 patients) for 4 weeks. The Glasgow Dyspepsia Severity Score (GDSS) was used to evaluate the relief of dyspepsia symptoms. Moreover, as a pilot secondary endpoint, a substudy (eight participants on TM and eight on placebo) was conducted in to evaluate gastric emptying (GE), estimated using a 99mTc-Tin Colloid Semi Solid Meal Scintigraphy test. *Results*: Of the 211 patients enrolled, 185 (87.7%) (97 (52.4%) in the TM group and 88 (47.6%) in the placebo group) completed the study and were analyzed. The groups did not differ in their demographic and medical history data. Regarding symptom relief, being the primary endpoint, a statistically significant reduction in GDSS for the TM group was revealed between the first (2-week) and final (4-week) visit (*p*-value = 0.02). The 99 mTc-Tin Colloid Semi Solid Meal Scintigraphy testing showed that TM significantly accelerated GE obtained at 50 min (median emptying 75.5% in the TM group vs. 66.6% in the placebo group, *p* = 0.036). Adverse effects of low to moderate severity were reported in 12.3% of the patients on TM. *Conclusion*: TM monotherapy appears to be an effective and safe approach to treating FD, although the findings presented here warrant further confirmation.

## 1. Introduction

The term “dyspepsia”, derived from the Greek words “*dys”* (bad) and *“pepsis”* (digestion), describes symptoms which originate from the upper gastrointestinal (GI) tract. Functional dyspepsia (FD), sometimes referred as idiopathic or nonulcer dyspepsia, is a bothersome and heterogeneous clinical syndrome affecting 10–40% [1] of the global population, influencing the quality of life of affected individuals and having substantial health and financial implications [2]. FD is characterized by abnormal sensations and peristalsis in the upper GI tract, in the absence of clinical syndromes of organic, metabolic or systemic disorders [3]. Although FD is one of the most common functional GI disorders, typically, only patients with persistent and/or recurrent problems consult a physician.

For a better comprehension and definition of functional gastrointestinal syndromes, the Rome Foundation introduced the internationally recognized and applied Rome diagnostic criteria. FD is characterized according to the third Rome revision by four cardinal symptoms: epigastralgia, bothersome postprandial fullness, epigastric burning and early satiety. FD patients are classified into two subgroups: epigastric pain syndrome (EPS) and postprandial distress syndrome (PDS). The last revision of Rome Criteria (IV) slightly updated the aforementioned definitions. Now, not only postprandial fullness, but also EPS symptom, as well as early satiation, should be considered as “bothersome symptoms”. Furthermore, the latest Rome IV classification implicated not only PDS and EPS as separate entities, but also the overlapping PDS and EPS; the latter syndrome is more common in the hospital-based population than among the general public [4].

The pathophysiology FD is poorly defined, leading to much uncertainty regarding therapeutic approaches. The proposed pathophysiologic mechanisms [5,6,7,8,9,10] involve altered GI motility, visceral hypersensitivity, including gastric hypersensitivity with distention of the stomach, impaired gastric accommodation of a meal, slow gastric emptying, altered duodenal sensitivity to certain nutrients found in food or acids, excessive acid secretion, *Helicobacter pylori* infection (*Hp*-I), nervous system dysregulation, low-grade inflammation, lifestyle influences, medication side effects, peripheral immune activation, genetic susceptibility and influences of socio-psychological factors (e.g., anxiety associated with postprandial distress syndrome, depression, introversion, stressful interpersonal relationships or stress). Nevertheless, the definite mechanism remains unidentified.

Since its pathophysiology is largely unknown, treatment of FD is difficult, and often yields unsatisfactory results. Camilleri and Tack [11] proposed that first line treatment be divided according to FD subtypes: Proton pump inhibitors (PPI) for EPS and prokinetics and/or 5-hydroxytryptamine (5HT) 1A agonist for PDS. If these treatments are not effective, PPIs can then be used for PDS, and prokinetics for EPS, as the second line treatment. Finally, in cases of no response to these drugs, antidepressants can be used.

Regarding current pharmacological and nonpharmacological therapeutic options for FD (including PPIs, *Hp* eradication, prokinetics, neuromodulators, dopamine-2 receptor antagonists, serotonin-4 receptor agonists, nonpharmacological regimens (e.g., physiotherapy, psychotherapy, acupuncture) and acotiamide (a muscarinic receptor antagonist)), relative evaluations concluded that treatments lack efficacy and/or safety, and for most therapeutic options, the precise mechanisms of action and true value in the treatment FD remain uncertain due to conflicting results [12]. Interestingly, except for the muscarinic receptor antagonist acotiamide, the authors of [12] note that trimebutine maleate (TM), a prokinetic drug, also exhibits antimuscarinic and mu, kappa and delta opiate receptors agonist effects, making it suitable for use in the treatment of many GI disorders [13]. It does not modify normal intestinal electrical activity in humans, but regulates abnormal hypo- or hyper- activity. Specifically, TM accelerates gastric emptying and shortens the lag period (i.e., the period before the onset of constant gastric emptying) [14]. It might also have possible antimicrobial properties against gastrointestinal bacteria that trigger postinfectious functional gastrointestinal disorders [14,15]. Importantly, almost all studies were conducted on TM and/or its combined regimens in irritable bowel syndrome (IBS) and/or FD overlapping disorders, but did not to evaluate TM monotherapy efficacy vs. placebo [14,16], TM vs. acotiamide, TM vs. mosapride or TM vs. domperidone [17]. TM, due to its mild side effects [18,19], can be substituted for other prokinetic agents that potentially exhibit severe opposing effects.

The aim of this study was to investigate, for first time, the efficacy and safety of TM as monotherapy for FD, given the present difficulties to sufficiently control the disorder pharmacologically. For this purpose, we conducted a multicenter, randomized, double-blind, placebo-controlled, prospective study comparing TM 300 mg BID (twice per day) vs. placebo, administered for a period of four weeks in patients diagnosed with FD.

## 2. Materials and Methods

A double-blind, randomized, placebo-controlled prospective trial was carried out in tertiary university hospitals at 13 sites in five countries (Greece, Turkey, Jordan, Poland and Romania) over a 65-month period (January 2011 to May 2016). Patients with FD as determined by the Rome III criteria, with exacerbated disease experiencing epigastric pain and/or discomfort at least one of the seven days before randomization, were candidates for enrollment. All participants provided written informed consent before enrollment, and the study protocol was approved by the medical ethics committee of the hospitals where the study was conducted (ethical code number 87/00-01/09, date of approval 29 June 2010).

Inclusion criteria were the following: (1) ambulant male and nonpregnant female subjects, (2) age between 18 and 75 years old at the randomization, (3) history of epigastric pain or discomfort at least one of the last seven days before the randomization, (4) absence of macroscopic findings from esophagus, stomach and duodenum during the endoscopy performed 16 ± 4 days before randomization, and (5) willingness to sign the informed consent form prior to entry into the study.

Exclusion criteria were the following: (1) alarm symptoms (loss of weight, vomit, hematemesis, melena, fever, jaundice or any other sign indicating serious or virulent disease), (2) IBS as defined by the Rome Criteria III, (3) chronic serious constipation, (4) predominantly retrosternal burning pain, (5) serious or uncontrolled diseases, (6) peptic ulcer disease (diagnosed by endoscopy or radiogram) or gastroesophageal reflux disease (diagnosed by endoscopy) and other significant GI disease, (7) history of peptic, gall-bladder or other abdominal surgery, (8) pregnancy or breastfeeding, (9) participation in a clinical trial within the previous month or current participation in any other clinical research study or clinical trial, (10) the detection of abnormal physical, hematology and biochemistry characteristics prior to randomization (at the investigator’s discretion), (11) usage of nonsteroidal anti-inflammatory drugs, (12) usage of oral contraceptives, (13) usage of platelet aggregation inhibitors, (14) usage of drugs that, from the investigators’ point of view, could cause dyspeptic symptoms, (15) alcoholism or psychological illness which could lead to noncompliance with the investigator’s instructions, and (16) known hypersensitivity to TM.

Three separate visits were scheduled. In the baseline visit (16 ± 4 days prior to randomization), basic laboratory testing was performed, and the absence of macroscopic endoscopic findings was confirmed. Demographic and medical data were also collected in this visit, and subsequently, the included participants were randomized to receive either TM 300 mg BID or placebo BID for four weeks. Two additional visits occurred at 2 and 4 weeks, according to the following parameters:

The primary efficacy endpoint of the study was relief of symptoms, which was evaluated by the Glasgow Dyspepsia Severity Scale (GDSS) [20], recorded at each study visit. A score reduction of at least three points was considered satisfactory. A GDSS of <2 was considered as complete clinical remission. Possible differences in GDSS reduction among the different participating countries were evaluated.

A secondary efficacy endpoint was the effect of treatment on patient quality of life, estimated using the Gastrointestinal Quality of Life Index (GIQLI) [21], recorded at each visit. A 50% improvement in quality of life was considered satisfactory. Another secondary efficacy endpoint was the measurement of time for gastric emptying and lag period via 99 mTc-Tin Colloid Semi Solid Meal Scintigraphy test, undertaken as a pilot substudy in the Greek site only by recruiting eight patients from the TM group and eight from the placebo group.

This test was performed and evaluated by an expert investigator (I.T.). The lag phase, T_1/2_ and Initial Activity score were recorded. Patients were instructed to fast and refrain from smoking overnight before the study. All 16 patients received a radiolabeled meal (Radiolabeled scrambled eggs, two slices of white toast bread (200 g) and a glass of water (250 mL) within 10 minutes); immediately afterwards, they were positioned at a γ-camera. One-min images were captured at 10-min intervals during the first hour and at 60, 120, 180 and 240 min with the patient standing. A computer-assisted method was used to determine the percentage of intragastric residual contents at 60, 120, 180, 240 min. The lag phase was determined either by a 5% drop in peak counts or by the first appearance of bowel activity. For the analyses, data were recorded as integrals (1–100) representing the percentage of activity retained in the stomach at various times. The duration of the lag phase, as well as the time required for the retained gastric activity to reach the 50% of the initial gastric activity, were estimated.

Throughout the study, and for 48 hours after administration of the last drug dose, TM safety evaluation included the recording of any adverse events, as well as their severity, their effect on the study drug and their management. All patients with adverse events were followed until symptoms resolved.

Descriptive statistics were used for demographic and medical data. Data are presented as mean ± standard deviation (SD) and numbers and/or percentages for continuous and categorical variables, respectively. Improvement vs. nonimprovement for patients receiving TM was also related to a list of variables through a logistic regression model. A paired *t*-test, after evaluating for normality, was performed in the analysis, undertaken according to protocol, within groups to test the reported relief from dyspeptic symptoms, graded using the GDSS. Comparisons of mean and median scores for each treatment group and between treatment groups were conducted. Also, patients who showed marked improvement were evaluated to determine whether there was a difference between those receiving TM and those receiving placebo.

A nonparametric Wilcoxon test was used to test median differences in the GDSS and GIQLI scores within each treatment group and between the two treatment groups. A secondary analysis tested whether the outcome measure was different in size between the participating countries using ANOVA, with the outcome measure as the primary response variable, the treatment as the explanatory factor and the country as the adjusting factor. A chi-square test with Yates correction was used to compare GDSS decrease proportion, complete symptom relief and the proportion of patients with adverse events, between the two treatment groups. Logistic regression analysis was used to correlate GDSS decrease with the demographic and medical data collected at the baseline visit, and also to analyze the two response variables for the quality of life results (more than 50% improvement in GIQLI scores and less than 50% improvement in GIQLI scores at the end of the study) where treatment, country and site were used as predictors. Logistic regression analysis was also used to analyze the proportion of recorded adverse events using treatment, severity, frequency, relation to medication and withdrawal due to treatment as predictors. Descriptive statistics analyses were used and confidence intervals were estimated for the reported differences in the estimated time for the retained gastric activity to reach 50% of the initial gastric activity using the 99 mTc-Tin Colloid Semi Solid Meal Scintigraphy test. Statistical significance was set at *p* < 0.05.

## 3. Results

**Figure 1:**
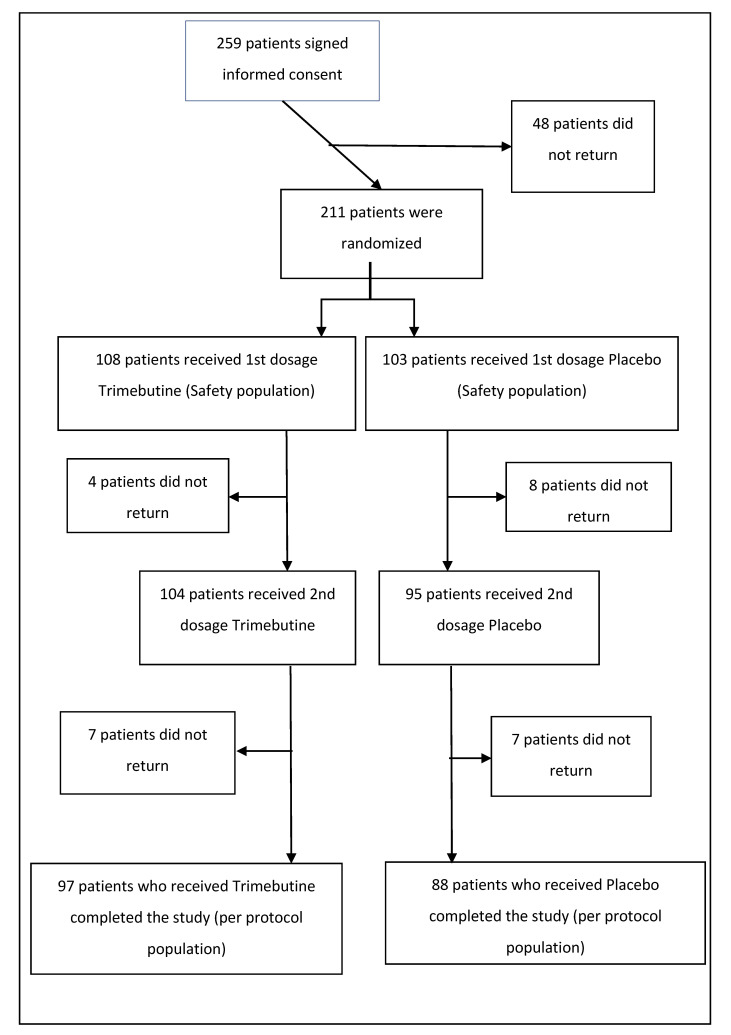
Flow chart of the experiment phase.

Two hundred and fifty-nine patients with FD were initially enrolled to participate in the study (Figure 1). A total of 211 eligible patients were considered for the recruitment phase and enrolled. Two groups were established to receive either TM 300 mg BID (108 patients—51.2%) or placebo (103 patients—48.8%). The patients returned for the first reevaluation two weeks after the initial treatment (104 or 52% on TM and 95 or 48% on placebo), where they completed all study assessments; 185 (87.7% of the randomized patients) patients again returned for the final (third, including baseline) assessment four weeks after the initial treatment (97 or 52.4% on TM and 88 or 47.6% on placebo). The basic demographics for the participants were as follows; regarding gender, 37 males (38.14%) and 60 females (61.86%) made up the TM group, while 35 males (39.77%) and 53 females (60.23%) made up the placebo group. Mean patient age was 41.2 (SD ± 13.7), median age was 40 and dominant age was 45 years of age. Concerning coexisting medical conditions, 23 patients (23.96%) in the TM group and 15 (17.44%) in the placebo group reported other symptoms. Moreover, three patients (3.09%) in the TM group and four (4.55%) in the placebo group had other abdominal conditions, whereas one patient (1.03%) in the former and four (4.55%) in the latter group were diagnosed with a dermatological condition. Finally, one patient in the TM group had a thyroid pathology (1.03%).

According to the nonparametric Mann-Whitney-Wilcoxon test, there was no difference in age by treatment (*p*-value = 0.81), height (*p*-value = 0.76) or weight (*p*-value = 0.73). With regard to patient medical history, 79% did not report any symptoms apart from dyspepsia, while 21% did. Patient examination proved normal for 98% of the patients. Sixty-five percent of the TM patients ate fried food, 50.5% ate salted food, 86.6% ate vegetables, 53.6% ate spicy food, whereas only 7.2% of patients had any food allergies. More than 25% occasionally consumed alcohol and 65% had never smoked, whereas 19.6% were regular smokers (Table 1).

Symptom relief compared to the baseline visit was evaluated as the primary endpoint by means of differences in GDSS. A statistically significant reduction of GDSS in the TM group was revealed between the first (2-week) and final (4-week) visit (*t*-test, *t*-value = 2.36, *p*-value = 0.02). The statistical difference remained robust, even after excluding patients who had GDSS equal to or less than 2 (complete symptom relief) (*t*-test, t-value = 1.94, *p*-value = 0.05).

Regarding 99mTc-Tin Colloid Semi Solid Meal Scintigraphy testing, as a secondary endpoint of the Greek pilot substudy, the following observations were made; TM significantly accelerated gastric emptying, i.e., occurring at 50 min (median emptying 75.5% TM group vs. 66.6% placebo group, *p* = 0.036), and shortened the lag period, albeit at an insignificant level (Lag Phage, *T_1/2_* (50%) 10.50 and 89.50 min, TM group vs. 12.50 and 90.00 min, placebo group, respectively).

Concerning the further secondary efficacy endpoint assessed by GIQLI questionnaires, although evident improvement was shown in more patients on TM, statistical significance was not obtained.

Additional results from this analysis include the following: More patients on TM showed improvement of symptoms and complete symptom relief in comparison to placebo, although the differences were not statistically significant. By constructing a logistic regression model with a list of independent variables for the patients on TM (dependent variable was the 3-point GDSS reduction), and separating patients into two groups (improvement vs. non improvement) (Table 2), only alcohol consumption was significantly associated with reduced probability of improvement (odds ratio: 0.219, *p =* 0.041).

Regarding the safety of TM, of the 211 patients who were randomized in the study, 26 (12.3%) reported adverse events (AE). Of those 26 patients, 15 (57.7%) were in the placebo group and 11 (42.3%) in the TM group. Of the 108 patients who received TM, 11 presented AE, representing a percentage of 10.2%. Similarly, of the 103 patients who received placebo, 15 presented AE (14.6%). These two percentages did not display a statistically significant difference. AEs were classified as mild, moderate or severe. AE reported in the TM group were mostly of mild intensity (nine mild and two moderate AE for TM group, compared to nine mild, six moderate and three severe AE for the placebo group). The placebo group included one AE case of severe headache and two cases of nausea. In only one of these cases, the drug was permanently discontinued whereas in remaining five AE cases, drug discontinuation was temporary (only one case in the TM group, i.e., 16.7%). Gastric testing was well tolerated in all Greek participants studied.

## 4. Discussion

In our prospective study, a potential benefit of TM was shown in a rather adequate patient sample following a randomized, placebo-controlled, double-blind design, which ensured the validity of the results. A primary efficacy outcome was achieved, with TM treatment being shown to be more effective in reducing GDSS than a placebo by the completion of the study. One of the two secondary efficacy outcomes, gastric emptying, as part of a smaller pilot substudy, was partly achieved, with TM significantly accelerating gastric emptying, i.e., within 50 min. Another study including patients with nonulcer dyspepsia, and probably with concomitant GERD disease, and healthy controls showed that an oral dose of 100 mg of TM three times daily for three weeks augmented gastric emptying rates with a concomitant decrease in lag period and less retention of food at 100 min (*p* < 0.0005). Specifically, when compared to pretreatment values, 17 patients exhibited accelerated gastric emptying, 2 showed no change, and 1 exhibited slower gastric emptying. The mean lag period after TM treatment was insignificantly slightly shorter than the lag period of controls (*p* > 0.05) and no significant difference was observed in the mean symptom score of patients with prolonged gastric emptying in the posttreatment period [22]. Due to the aforementioned inconclusive data, further large-scale relative studies are needed to elucidate these points.

Overall, in our study, TM was shown to be a safe treatment choice with only mild to moderate adverse events, which were easily manageable.

A plethora of interesting relevant studies have investigated the effect of TM and other agents on FD and related pathologies. One of the very first studies evaluating the efficacy of TM was performed by Lüttecke et al. [23]. Briefly, three controlled studies took place, which included IBS patients with symptoms of dyspepsia, abdominal distension, constipation, diarrhea, pain and flatulence. The administration of 200 mg TM three times per day for a total of three days in the first two trials was shown to significantly reduce the aforementioned symptoms compared to the placebo group. This significance was, however, lost once the TM dosage was halved. In the last experiment, it was shown that a 200 mg TM dosage for half a month was as efficient as mebeverine (100 mg 4 times per day). The authors noted that no serious TM-associated side effects were seen.

Zhong et al. [19] examined the efficacy, as well as the possible adverse effects, of TM in patients suffering from FD with accompanying IBS (diarrhea dominant). For this purpose, 129 patients were prospectively enrolled, and after randomization, were divided into three groups; TM and *Bacillus licheniformis*, TM alone, and *Bacillus licheniformis* alone. Statistically significant decreases were reported in the scores of postprandial fullness, early satiation, abdominal pain and total symptoms for the first two groups before and after treatment, whereas there was no effect for the third group. A significant decrease in the diarrhea score before and after treatment in all groups was also documented. After treatment for one month, significant differences in the scores of early satiation, postprandial fullness, abdominal pain and total symptoms, as well as the effective rate of each symptom and total effective rate between first or second and third group, were exhibited. The authors deduced that treating patients with the aforementioned profile with TM appeared to have the advantage of high efficacy, low cost and low likelihood of adverse reactions [19].

Furthermore, in an interesting pediatric study, a total of 345 children and adolescents with IBS diagnoses (according to Rome III criteria) were recruited. The prevalence of FD was 80% for the IBS group. Half of the patients were treated with TM for three weeks and the other half with a placebo. A statistically significant difference in clinical recovery was noted between the groups (94.9% TM group vs. 20.5% placebo group) [24].

Prokinetic agents have shown a beneficial effect in FD, as confirmed in a meta-analysis of all studies involving prokinetic drugs [25]. Fourteen trials, including 1053 patients, were identified (all but one trial tested cisapride, a mixed 5HT4 agonist and 5HT3 antagonist). The outcome was classified as dyspepsia improvement or lack; a significant decrease in dyspepsia was observed with prokinetic therapy vs. placebo (relative risk 0.52; 95% CI, 0.37–0.73). However, the studies were highly heterogeneous, casting some doubt on the actual effect size, and cisapride has been withdrawn from the US and other global markets due to its cardiac toxicity. The efficacy of domperidone in FD, which is also unavailable in the USA (except as an investigational drug) but prescribed elsewhere, remains uncertain, as high quality trials have not been undertaken. Although PPIs seem to have an effect in cases where pain and heartburn predominate, prokinetics seem to be more effective in cases where nausea, postprandial fullness and early satiety predominate.

In a further meta-analysis (Bayesian network) [26] involving prokinetics for the treatment of FD, 25 randomized control trials were included with a total of 4473 FD patients who were treated with six different prokinetics or placebo. The authors concluded that TM, metoclopramide, domperidone and mosapride showed better efficacy for the treatment of FD than itopride or acotiamide.

Stanghellini et al. [27] examined gastric emptying in 146 IBS patients using scintigraphy, as well as dyspeptic symptoms using a validated questionnaire. Dyspepsia was found in 96 (66%) patients, with patients complaining of postprandial fullness (88%), epigastric pain (48%), nausea (30%) and vomiting (9%). They found a higher prevalence of abnormal gastric emptying in patients with IBS and dyspepsia compared with those with only IBS, in both men and women. Gastric emptying in IBS overlapping dyspepsia was significantly delayed compared to IBS without dyspepsia or healthy controls. In this respect, recent data indicate that regardless of the gastric emptying delay by using scintigraphy, patients with FD symptoms exhibit a high prevalence of esophageal motor disorder and abnormal esophageal acid exposure that might contribute to their symptoms, thereby requiring therapy [28]. TM may exhibit a protective effect against gastroesophageal reflux disease (GERD) [13]. Moreover, published trials have shown that promotility mediators can considerably accelerate gastric emptying (when optimal scintigraphy methods were introduced), producing noteworthy improvements in FD symptoms [29].

There is emerging evidence that FD coexists as an overlapping syndrome with IBS and GERD. Such patients are characterized by a worse health-related quality of life (HR-QOL) score. Moreover, *Hp-*I has been as regarded as contributor in the pathogenesis, at least partly, of these overlapping entities [14,16]. Our preliminary data have demonstrated [13] that *Hp*-I is frequent in patients with GERD-IBS-FD and/or erosive esophagitis, and that *Hp* eradication, along with PPIs and/or TM regimens, offer improvement of HR-QOL, predominantly in patients treated with TM [13]. Mechanistically, *Hp*-induced mast cells are regarded as important effectors of the gut–brain axis that translate stress signals into the induction of variable neurotransmitters and proinflammatory mediators that might contribute to GI tract pathophysiology. *Hp*-stimulated chronic perceived stress results in decreased host defense and initiates intestinal inflammation through mast cell-dependent mechanisms, thereby signifying the activation of peripheral corticotropin-releasing factor receptors (CRF-Rs) and mast cells as significant mechanisms involved in stress-linked GI pathophysiology [14,16]. In this regard, recent data indicate that mast cell activation seems to play a role in FD pathophysiology [30]. Specifically, CRF_2_ appears to play an essential role in gastric hyperalgesia induced by stress which evokes mast cell degranulation; thus, CRF_2_ signaling could be a valuable therapeutic target for FD [31]. Likewise, impaired gastric barrier function occurs in FD patients, and its increased permeability is correlated with mast cell quantities of FD patients, thereby providing an additional novel pathophysiological mechanism and therapeutic target for the treatment of FD [32].

Regarding preclinical studies, TM was recently shown to be effective at maintaining upper and lower GI motor function in a peripheral CRF overlapping model with guinea pigs [33]. It is worth noting that apart from GI tract motility disorders, recent data suggest that TM may exert antitumor activities against gut and brain malignancies [34].

Despite its adequate number of participants, our study was also characterized by several limitations which necessitate further research; we have to acknowledge that the attrition rate was significant for both groups, being over 10%. Moreover, there was no available data in the literature regarding a validated cutoff value of GDSS. We therefore assumed that a decrease of at least three points in the GDSS would clinically reflect a reasonable level of relief of FD symptoms. Furthermore, we did not use the latest Rome IV criteria, since our study was initiated before the introduction of the new criteria. Moreover, the long-term efficacy of TM in FD treatment was not evaluated; as in previous studies, we were constrained to four-week period, noting the early onset of antispasmodic efficacy in this time frame [35]. Our analysis was also undertaken according to protocol and not with intention-to-treat. Furthermore, we did not evaluate *Hp* status as a potential contributor to the pathophysiology of the studied patients. The main reason for this was the obvious bias attributable to the high rate of false negative results due to the study’s design, which excluded patients with macroscopic findings from esophagus, stomach and duodenum during the endoscopy; endoscopic abnormal patterns such as “mosaic-like” appearance, “diffuse homogeneous redness” and/or “irregular redness with groove,” predict the presence of *Hp*-I infection with a sensitivity of 93.3 %, a specificity of 89.1 %, and an overall accuracy of 91.6 % in patients with any of the “abnormal” patterns [36]. Since TM also displays antibacterial properties [14,15], in the future, it would be of great interest to evaluate whether it could serve as an adjuvant in *Hp*-*I* eradication. Moreover, distinctions were not made among PDS, EPS and PDS–EPS overlap syndromes. Lastly, a meal scintigraphy test was performed as a pilot substudy in one center, involving a small number of participants. Thus, further, large-scale prospective studies are required to confirm our preliminary data regarding its potential objective value in estimating the effect of TM in FD patients.

## 5. Conclusions

Within this multicenter, randomized, double-blind, placebo-controlled, prospective study, a sufficient number of patients was recruited. It was demonstrated for the first time that TM monotherapy is an effective and safe option for the treatment of FD.

All in all, this study might provide further insights into the treatment of FD, which is, at present, inadequate, despite the amount of knowledge gathered in recent years about the condition, in particular, with regards to its etiologic associations (e.g., the minor role of *Hp*) and the relationship between symptoms and pathophysiologic mechanisms [37].

Further large-scale randomized control trials are required to verify the safety and efficacy of TM, which might lead to the inclusion of this promising pharmaceutical agent in future guidelines.

## Figures and Tables

**Table 1:** Diet and habits.

Variable	Trimebutine *N* (%)	Placebo *N* (%)	*p*-Value
**Diet**			
Fried Food	63 (64.95%)	50 (57.47%)	0.36
Salted Food	49 (50.52%)	50 (57.47%)	0.38
Vegetables	84 (86.6%)	70 (80.46%)	0.32
Spicy Food	52 (53.61%)	43 (50%)	0.66
Food Allergies	7 (7.22%)	9 (10.34%)	0.6
**Smoking History**			
Nonsmoker	63 (64.95%)	59 (67.05%)	0.82
Previous Smoker	9 (9.28%)	5 (5.68%)	
Occasional Smoker	6 (6.19%)	5 (5.68%)	
Regular Smoker	19 (19.59%)	19 (21.59%)	
Alcohol	24 (25%)	25 (29.07%)	0.62

**Table 2:** Effects of independent variables on the probability of improvement.

	*B*	*S.E.*	*Wald*	*df*	*Sig.*	*Exp(B)*
Sex	0.051	0.677	0.006	1	0.940	1.052
Age	−0.028	0.029	0.929	1	0.335	0.973
BMI	−0.075	0.089	0.716	1	0.398	0.927
History of operations	0.599	0.639	0.880	1	0.348	1.820
Smoking	−0.204	0.608	0.113	1	0.737	0.815
Alcohol consumption	−1.519	0.744	4.169	1	0.041	0.219
Fried food	−1.534	0.814	3.553	1	0.059	0.216
Salted food	0.667	0.674	0.979	1	0.322	1.948
Vegetables	1.599	0.909	3.099	1	0.078	4.950
Spicy food	−0.252	0.697	0.131	1	0.718	0.777
Country of residence	0.461	0.311	2.201	1	0.138	1.585
